# Correlation between septal midwall late gadolinium enhancement on CMR and conduction delay on ECG in patients with nonischemic dilated cardiomyopathy

**DOI:** 10.1016/j.ijcha.2020.100474

**Published:** 2020-01-25

**Authors:** Marthe A.J. Becker, Cornelis P. Allaart, Alwin Zweerink, Jan H. Cornel, Peter M. van de Ven, Albert C. van Rossum, Tjeerd Germans

**Affiliations:** a Amsterdam UMC, Vrije Universiteit Amsterdam, Department of Cardiology, Amsterdam Cardiovascular Sciences, De Boelelaan 1117, 1081 HV Amsterdam, the Netherlands; b North West Hospital Group Alkmaar, Department of Cardiology, Wilhelminalaan 12, 1815 JD Alkmaar, the Netherlands; c Radboud University Medical Center, Department of Cardiology, Geert Grooteplein Zuid 10, 6525 GA Nijmegen, the Netherlands; d Amsterdam UMC, Vrije Universiteit Amsterdam, Department of Epidemiology and Biostatistics, De Boelelaan 1117, 1081 HV Amsterdam, the Netherlands

**Keywords:** QRS duration, Conduction delay, Septal midwall LGE, Nonischemic dilated cardiomyopathy

## Abstract

**Background:**

Septal midwall late gadolinium enhancement (LGE) on cardiac magnetic resonance imaging (CMR) is a characteristic finding in nonischemic dilated cardiomyopathy (DCM) and is associated with adverse cardiac events. QRS-prolongation in DCM is also frequently present and a predictor of arrhythmic events and mortality. Since the His-Purkinje fibres are located in the interventricular septum, QRS-prolongation may directly result from septal fibrosis, visualized by LGE. Our aim was to study the correlation of the presence and extent of septal midwall LGE and QRS-duration.

**Methods:**

DCM-patients with left ventricular (LV) dysfunction (LVEF < 50%) were included. LV volumes, systolic function and nonischemic septal midwall LGE, defined as patchy or stripe-like LGE in the septal segments, were quantified. QRS-duration on standard 12-lead ECG was measured.

**Results:**

165 DCM-patients were included (62% male, mean age 59 ± 15 years) with a median LVEF of 36% [24–44]. Fifty-one patients (31%) demonstrated septal midwall LGE with a median extent of 8.1 gram [4.3–16.8]. Patients with midwall LGE had increased LV end-diastolic volumes (EDV) 248 mL [193–301] vs. 193 mL [160–239], p < 0.001) and lower LVEF (26% [18–35] vs. 40% [32–45], p < 0.001). Median QRS-duration was 110 ms [95–146] without a correlation to the presence nor extent of midwall LGE. QRS-duration was moderately correlated with LV-dilation and mass (respectively r = 0.35, p < 0.001 and r = 0.30, p < 0.001).

**Conclusion:**

In DCM-patients, QRS-prolongation and septal midwall LGE are frequently present and often co-exist. However, they are not correlated. This suggests that the assessment of LGE-CMR has complementary value to ECG evaluation in the clinical assessment and risk stratification of DCM-patients.

## 1 Introduction

In patients with heart failure with reduced left ventricular ejection fraction (LVEF), cardiac magnetic resonance imaging (CMR) using late gadolinium enhancement (LGE) is able to distinguish an ischemic and nonischemic aetiology in a non-invasive manner [1], [2]. A typical finding on CMR in patients with nonischemic dilated cardiomyopathy (DCM) is septal midwall LGE, whereas a subendocardial or transmural contrast pattern is more characteristic for an ischemic cause [2]. Moreover, the presence of midwall LGE in patients with DCM heralds a poor prognosis regarding survival, symptomatic heart failure or ventricular arrhythmic events including ventricular tachycardia or fibrillation and sudden cardiac death, irrespective of LV function [3], [4], [5]. In addition, the absence of midwall LGE in patients with DCM is associated with functional recovery of systolic dysfunction [4], [6]. However, CMR is not widely available.

QRS-prolongation on electrocardiography (ECG) with or without mechanical dyssynchrony is frequently observed in DCM and the presence is associated with LV dysfunction and is predictive for cardiovascular adverse events during follow-up [7], [8], [9], [10]. The LV dysfunction due to QRS-prolongation with mechanical dyssynchrony may be potentially reversible with resynchronization therapy, especially in patients with QRS-prolongation due to left bundle branch block (LBBB). However, if midwall LGE is present on CMR, functional recovery is less likely [5]. We hypothesized that septal midwall LGE on CMR in patients with DCM, which visualizes the interstitial fibrotic changes due to adverse remodelling in heart failure [11], [12], directly affects the conduction system located in the interventricular septum resulting in an altered electrical activation pattern with QRS-prolongation. We aimed to characterize the relation between ventricular conduction and septal midwall fibrosis in DCM-patients with LGE-CMR.

## 2 Methods

Patients with symptomatic heart failure and a decreased left ventricular systolic function defined as LVEF < 50% who underwent LGE-CMR between 216 and 2018 were included in this observational study. Dilated cardiomyopathy was classified according to current guidelines [13] as LV dysfunction in the absence of significant obstructive coronary artery disease at anatomical or functional imaging. Moreover, a history of myocardial infarction or revascularization and ischemic LGE-patterns on CMR (defined as subendocardial or transmural in a coronary artery territory) of sufficient severity to explain the degree of dysfunction was also excluded. Furthermore, patients with LV dysfunction attributed to severe systemic hypertension, primary valvular heart disease, congenital heart disease, acute myocarditis, hypertrophic cardiomyopathy or arrhythmogenic cardiomyopathy were excluded as well as patients with pacemaker or ICD. The investigation conforms with the principles outlined in the Declaration of Helsinki. Patients provided written informed consent for data collection. The local ethics review committee approved the data collection and management of this study.

DCM patients underwent CMR imaging either for assessment of underlying aetiology in newly developed heart failure or for prognostic evaluation prior to ICD-implantation. Scans were performed on 1.5 Tesla whole body scanners (Siemens, Erlangen, Germany and GE Heathcare, Chicago IL, United States of America) with dedicated phased array cardiac receiver coils. Cine imaging was performed using retrospective ECG-gated steady-state free precession cine during breath hold in standard 3 long-axis views and a stack of short-axis slices, covering the ventricles from base to apex.

LGE images were acquired 10–15 min after gadolinium contrast administration using a T1-weighted inversion recovery-prepared gradient echo sequence with optimized inversion time. The presence and pattern of gadolinium hyperenhancement were assessed visually by certified CMR-cardiologists and LGE was considered present if the enhancement was seen in two perpendicular views or two serial slices. The septal midwall LGE pattern was defined as stripe-like or patchy midmyocardial hyperenhancement in the interventricular septal segments. Endocardial contours were manually drawn in end diastolic and end systolic phase on a stack of short axis cine images, using dedicated software (CVI^42^ Circle Cardiovascular Imaging Inc., Calgary, Canada). From that dataset, left ventricular end diastolic volume (LVEDV), end systolic volume (LVESV) and LVEF were calculated. The extent of LGE was quantified using the full width at half maximum (FWHM) method [14]. LV endo- and epicontours were delineated at short-axis LGE-images. Secondly, a region of interest was selected in the hyperenhanced myocardium to define maximum signal intensity for the FWHM-threshold. The basal short axis slice with the LV outflow tract visible was excluded from analysis and obvious blood pool, pericardial fat or artefacts were excluded manually.

A resting 12-lead electrocardiogram (ECG) of each patient, made in supine position (0.5–150 Hz, 25 mm/s, 10 mm/mV), was collected. Heartrate (beats per minute (bpm)) was automatically assessed. Measurements included the PR-interval and QRS duration, assessed manually from onset of first deflection from baseline (either negative deflection of a Q-wave, or positive deflection of the R wave) until the end of the S-wave, defined as its return to baseline. Left bundle branch block (LBBB) was defined according to conventional criteria of AHA/ACCF/HRS [15], including QRS duration ≥120 ms with monophasic QS or rS-complex in V1, a broad, frequently notched R wave in lateral leads I, aVL, V5 or V6, and absent Q-wave in V5-V6. When QRS duration was >120 ms but did not fulfil criteria for LBBB, it was classified as non-LBBB. In patients with atrial fibrillation, only heartrate and QRS duration were assessed. Median duration between CMR and ECG was 1 week [−1, 7].

Continuous variables are presented as mean ± SD, or as median and interquartile range (IQR), depending on whether distribution was normal or not. Categorical data are summarized as frequencies and percentages. The independent samples *t*-test or Mann-Whitney *U* test were used for comparison between groups for continuous data, depending on normality, and chi-square test for intergroup comparison of binomial data. Mann-Whitney *U* test was also used for the comparison of ordinal categorical variables. Pearson and Spearman correlations were calculated as a measure of association between LV volumes, LGE amount and QRS duration, depending on normality. For all these analyses a p-value of <0.05 was considered statistically significant. Determinants of septal midwall LGE and QRS-duration was assessed using respectively logistic regression analysis and linear regression analysis. Data included in regression analysis included demographic data and CMR-derived variables. Parameters with p < 0.1 in univariable regression models were entered in a multivariable regression analysis using a backward elimination procedure with p < 0.05 required for inclusion in the final model. R square and Nagelkerke R were used to assess goodness-of-fit of the linear and logistic regression model. Collinearity was assessed for multivariable analysis using variance inflation factors. No imputation of missing data was performed. Analyses were performed using SPSS (version 24, IBM SPSS Statistics, Chicago, IL, USA).

## 3 Results

We included 165 patients with DCM. Table 1 depicts the patient characteristics, stratified by the presence or absence of septal midwall LGE. The majority was male (62%), mean age was 59 ± 15 years. Mean heartrate on ECG was 74 ± 15 bpm and most patients had sinus rhythm (89%). Median LVEF was 36% [26–46] and LGE was present in 62 patients (38%) with a median extent of 8.1 g [4.9–20.7]. Septal midwall LGE was found in 51 patients (31%) and the median extent was 8.1 g [4.3–16.8]. Eleven patients had LGE in locations other than septal midwall; these included focal ischemic patterns or patchy enhancement in the lateral or inferior wall.

**Table 1:** Patient characteristics.

	Total	Septal midwall LGE	No septal midwall LGE	p-value
Number	165	51	114	
Male sex	103 (62%)	36 (71%)	67 (59%)	0.15
Age (year)	59 ± 15	62 ± 13	57 ± 15	0.06
BMI (kg/m^2^)	26 ± 5	26 ± 5	26 ± 5	0.77
NYHA class				0.02†
Class I	57 (35%)	11 (22%)	46 (40%)	
Class II	49 (30%)	20 (39%)	29 (25%)	
Class III/IV	33 (20%)	13 (26%)	20 (18%)	
Unknown	26 (16%)	7 (14%)	19 (17%)	

**CMR**				
LVEDV (mL)	211 [172–267]	248 [193–301]	193 [160–239]	<0.001
LVEDVi (mL/m^2^)	109 [88–133]	128 [106–154]	99 [83–121]	<0.001
LVESV (mL)	131 [101–190]	182 [125–241]	115 [97–153]	<0.001
LVESVi (mL/m^2^)	67 [50–98]	92 [65–124]	59 [47–78]	<0.001
LVSV (mL)	68 [55–82]	62 [46–77]	72 [57–92]	0.03
LVEF (%)	36 [24–44]	26 [18–35]	40 [32–45]	<0.001
LV mass (grams)	150 ± 46	169 ± 52	142 ± 40	<0.001
Total LGE extent (gram)	8.1 [4.9–20.7]	8.4 [6.0-23.1]	4.2 [2.7-10.9]	0.01
Septal midwall LGE extent (gram)	8.1 [4.3–16.8]	8.1 [4.3–16.8]	–	–

**Medication**				
B-blocker	111 (67%)	41 (80%)	70 (61%)	0.02
ACEi or ARB	118 (72%)	43 (84%)	75 (66%)	0.02
MRA	55 (33%)	28 (55%)	27 (24%)	<0.001
Diuretics	65 (39%)	28 (55%)	37 (33%)	0.01

**ECG**				
Sinus rhythm	147 (89%)	45 (88%)	102 (90%)	0.81
HR (bpm)	74 ± 15	75 ± 13	73 ± 16	0.37
AV-delay (ms)	176 ± 31	187 ± 37	171 ± 27	0.003
1st degree AV-block	21 (13%)	9 (18%)	12 (11%)	0.19
QRS duration (ms)	110 [95–146]	104 [95–151]	114 [95–146]	0.76
QRS < 120 ms	91 (55%)	30 (59%)	61 (54%)	0.53
LBBB	56 (34%)	12 (24%)	44 (39%)	0.06

ACEi: angiotensin-converting enzyme inhibitor, ARB: angiotensin-II receptor blocker, AV: atrioventricular, bpm: beats per minute, BMI: body mass index, CMR: cardiac magnetic resonance imaging, DCM: dilated cardiomyopathy, ECG: electrocardiogram, HR: heart rate, LBBB: left bundle branch block, LV: left ventricle, LVEF: left ventricular ejection fraction, LGE: late gadolinium enhancement, LVEDV(i): left ventricular end-diastolic volume (index), LVESV(i): left ventricular end-systolic volume (index), LVSV: left ventricular stroke volume, MRA: mineralocorticoid receptor antagonist, ms: milliseconds, NA: not applicable, NYHA: New York Heart Association functional class within 1 month of CMR.

† Patients with unknown NYHA functional class were excluded from analysis.

**Fig. 1:**
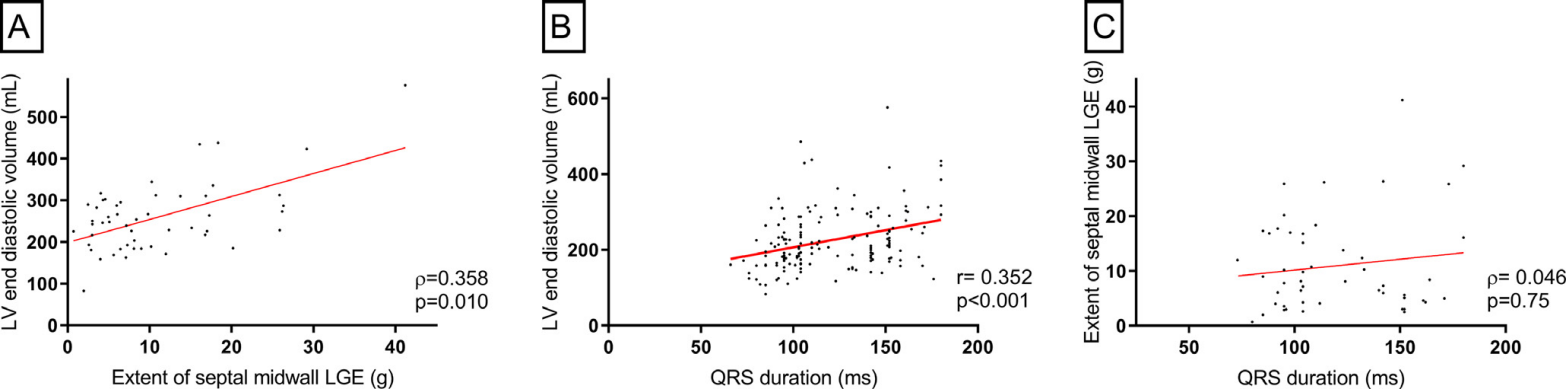
Correlation between QRS-duration, LGE-extent and LV dilation. Both the extent of septal midwall LGE (A) and QRS-duration (B) were moderately correlated to LV end diastolic volume. However, QRS duration and LGE-extent showed no significant correlation at all (C). Abbreviations: g: grams, LGE: late gadolinium enhancement, LV: left ventricular. mL: millilitre, ms: milliseconds. Symbols: ρ: Spearman correlation coefficient, r: Pearson correlation coefficient.

Patient with septal midwall LGE had significantly more severe LV dilation (median LVEDV 248 mL [194–302] vs. 193 mL [154–233], p < 0.001) and worse LV systolic function (LVEF 26% [18–35] vs. 40% [32–45], p < 0.001) compared to patients without septal LGE (Table 1). LGE-extent showed a moderate correlation only to LV volumes (LVEDV r = 0.358, p = 0.010, Fig. 1A) and to LV mass (r = 0.395, p = 0.004). Determinants of the presence of septal midwall LGE at univariable analyses were age (OR 1.02 per year [95% CI 1.00–1.05], p = 0.08), NYHA functional class, (OR for class II 2.88 [1.21–6.89], p = 0.02, and OR for class III-IV 2.72 [1.04–7.09], p = 0.04), LVEDV (OR per 10 mL 1.09 [1.04–1.14], p = 0.001), LVEF (OR per 10% 0.47 [0.34–0.64], p < 0.001), and LV mass (OR 1.01 [1.01–1.02], p = 0.001).

The mean PR-interval was 176 ± 15 ms and was significantly longer in patients with midwall LGE present (187 ± 37 ms vs. 171 ± 27 ms, p = 0.003 (Table 1)). However, length of PR-interval showed no association with extent of septal midwall LGE (r = 0.16, p = 0.30).

**Fig. 2:**
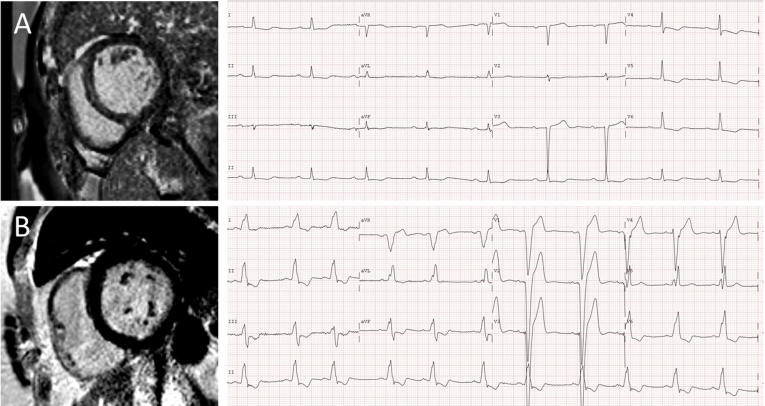
Case examples of LGE-CMR imaging and 12-lead ECG. Case example of two DCM-patients, one (A) with septal midwall LGE on CMR, however on ECG a first degree AV-block and a small QRS complex and the other (B) without LGE on CMR with a typical LBBB on ECG. Abbreviations: CMR: cardiac magnetic resonance imaging, DCM: dilated cardiomyopathy, ECG: electrocardiogram, LBBB: left bundle branch block, LGE: late gadolinium enhancement.

Median QRS duration was not found to differ between patients with and without septal midwall LGE (respectively 104 ms [95–151] vs. 114 ms [95–146], p = 0.79). The majority of the 74 patients with QRS ≥ 120 ms had an LBBB pattern (76%). The prevalence of LBBB did not differ significantly between patients with and without septal midwall LGE (24% vs. 39%, p = 0.06) and neither did the prevalence of narrow QRS-complex (59% vs. 54%, p = 0.53). We found a moderate positive correlation between QRS duration and LV dilation, expressed as LVEDV (r = 0.35, p < 0.001. Fig. 1B) and between QRS duration and LV mass (r = 0.30, p < 0.001). Only a weak negative correlation to LVEF (r = −0.27, p < 0.001) was found. There was no significant correlation between QRS duration and presence nor extent of septal LGE (r = 0.05 and p = 0.75, Fig. 1C; case example Fig. 2). Moreover, total LGE extent and QRS duration were not correlated either (r = 0.05, p = 0.69). Associates of QRS-prolongation are presented in Table 2. Included in the final multivariable model were age (B 0.53 per year increase [0.22–0.85], p = 0.001) and LV mass (B 0.22 per g increase [0.12–0.32], p < 0.001). NYHA-functional class, LVEDV and LVEF, all significant in univariable analysis, were removed from the model in the backward elimination procedure (Table 2).

**Table 2:** Association with QRS duration.

	Univariable linear regression			Multivariable linear regression		
	B	95% CI	p-value	B	95% CI	p-value
Male sex	0.41	−8.81 to 9.63	0.93			
Age (year)	0.57	0.28–0.86	<0.001	0.53	0.22–0.85	0.001
BMI (kg/m^2^)	0.07	−0.88–1.01	0.89			
NYHA functional class						NS
Class I	1.00	–	(ref)			
Class II	11.22	0.13–22.31	0.05			
Class III/IV	2.51	−9.94–14.96	0.69			
LVEDV per 10 mL	1.24	0.70–1.77	<0.001			NS
LVEF per 10%	−5.88	−9.59 to −2.14	<0.01			NS
LV mass (g)	0.21	0.12–0.31	<0.001	0.22	0.12–0.32	<0.001
Total LGE extent (g)	0.15	−0.59–0.89	0.69			
Septal LGE presence	−1.58	−11.25–8.08	0.75			
Septal LGE extent (g)	0.48	−0.51–1.47	0.34			
Total LGE extent (g)	0.15	−0.59–0.89	0.69			
Heart rate (bpm)	−0.27	−0.56–0.02	0.07			NS
AV-delay (ms)	0.06	−0.10–0.21	0.49			

Table presents the association between patient characteristics and QRS duration. NS indicates a parameter that was included as candidate predictor in multivariable analysis, but removed in backward selection procedure and therefore does not appear in the final model (i.e. nonsignificant). CI: confidence interval, LV: left ventricle, LVEDV: left ventricular end-diastolic volume, LVEF: left ventricular ejection fraction, NYHA: New York Heart Association functional class.

## 4 Discussion

In the present study, both septal midwall LGE on CMR and QRS-prolongation on ECG were frequently observed and often co-existed in patients with DCM. Although both parameters were significantly related to the extent of LV dilation and severity of LV systolic dysfunction, ventricular conduction delay was not correlated to the presence nor the extent of septal midwall LGE.

In patients with cardiomyopathy, left ventricular remodelling occurs to maintain cardiac output and is accompanied by myocyte hypertrophy and an increase of interstitial fibrosis [12], [16], [17]. In long term persistent heart failure, the normally well-balanced cascades of production and degradation of extracellular matrix are disrupted and the initially reversible interstitial fibrosis may progress into irreversible replacement fibrosis [17], [18]. This results in an increase of extracellular space, which can be visualized by LGE, although this depends on focal differences [18], [19]. The pattern of septal midwall LGE has been described primarily in patients with DCM [2] and demonstrated good agreement with septal myocardial fibrosis in previous histopathological studies [20], [21].

The present study showed that in the presence of midwall LGE, LV volumes were larger, LV mass was increased and LV function was significantly lower. This is in line with earlier studies [5], [22], and strongly suggests that the presence of midwall LGE represents an advanced stage of myocardial remodelling. This apparent association between septal midwall LGE presence and the advanced stage of remodelling may also explain why the presence of LGE indicates poor prognosis in DCM patients in terms of mortality, heart failure and ventricular arrhythmic events [3], [5], [23].

Interstitial fibrosis is assumed to reduce conduction velocity [16]. Interestingly, we did not find an association between QRS duration and septal midwall LGE presence or extent in our study. The present findings are in contrast to the recent study of Grigoratos et al. [24], where QRS-prolongation and LBBB were significantly correlated to septal scar-extent. However, the cut-off for LGE-quantification differed from our study, and septal scar was not specified, which may have included ischemic scar. The difference may be explained by the observation that Purkinje-fibres are located in the subendocardium and not mid wall [25], [26]. Purkinje fibres are typically affected and disrupted during ischemia, explaining QRS-prolongation during ischemia [27] and in ischemic fibrosis [28]. In DCM, interstitial fibrosis mainly occurs in the mid myocardium amidst the myocardial fibres [12], thereby apparently sparing the subendocardium and conduction fibres. This is in line with a previous study who found that midwall LGE and LBBB were independent predictors of prognosis [5].

QRS-prolongation with LBBB is found in approximately 1% of the general population and up to 30% of heart failure patients. The prevalence of LBBB is increasing with age, and in patients with structural heart disease [8], [24], [29]. The resultant dyssynchronous contraction pattern induces reduction of global LV function, which was found to result in the development of heart failure in one third of patients during long-term follow-up [29], [30], [31]. Previous studies demonstrated that LV function may recover after resynchronization of mechanical dyssynchrony [29], [31], in particular if LBBB is present and QRS duration is ≥150 ms [32]. In contrast, concomitant septal midwall LGE on CMR in DCM-patients was associated with poor response to resynchronization therapy by biventricular pacing [5], underscoring the interrelation between septal midwall LGE and irreversible LV remodelling irrespective of the presence of LBBB.

LV dilation, resulting from prolonged LV remodelling in heart failure, was moderately correlated to QRS-prolongation in the present study. This finding is in line with previous work [33], [34] and has been attributed to the increased path-length of the conduction system. Whereas QRS duration was not related to LGE, the AV-delay in our cohort, expressed as PR-interval, was increased in the presence of septal midwall LGE, although without significant correlation to the extent of LGE. This may be the result of often concomitant LA dilatation which frequently occurs in DCM patients [35]. A histopathologic study showed anatomical elongation of the AV bundle, causing increased path lengths as part of the remodelling process, resulting in delayed AV conduction in heart failure [36].

This study demonstrates that QRS-prolongation and septal midwall LGE are different entities in patients with DCM. Since all these findings are individually associated with structural changes in LV dimension and function, and all have prognostic consequences [3], [5], [8], combining these risk factors might enhance risk stratification in DCM-patients. A recent study showed that combining LGE-CMR and QRS-prolongation for the prediction of mortality and ventricular arrhythmias reached a higher diagnostic accuracy than LGE-CMR alone [22]. As both determinants are often routinely obtained during assessment of heart failure patients, they are readily available for improvement of risk stratification of DCM patients. Further research in this respect is warranted.

## 5 Limitations

There are several limitations to this study. This was a single-centre, retrospective, observational study, which is associated with potential selection bias and the rather small sample size limits the power of these results. Furthermore, LGE-quantification was performed after visual assessment of presence or absence of septal midwall LGE. However, the interobserver variability of a certified cardiologists assessing the presence of midwall LGE is known to be small [3]. In addition, LGE-quantification remains a rather subjective and variable analysis, in particular of nonischemic LGE-patterns. However, we used the full-width half maximum technique, which provided the best reproducibility in nonischemic LGE-pattern in a previous study [14]. Thirdly, the relationship between QRS duration and diffuse interstitial fibrosis could not be assessed in this study, since diffuse fibrotic changes cannot be visualized by LGE-CMR. This limitation can be overcome by the assessment of diffuse interstitial fibrosis using T1-mapping and extracellular volume (ECV) quantification. A recent meta-analysis demonstrated that both native T1-mapping and ECV can differentiate between cardiomyopathy patients and healthy controls, with significantly increased native T1-values and ECV in patients with DCM [37]. However, the retrospective nature of this study limited these analysis, considering that T1-mapping is not routinely performed in clinical CMR-analysis of patients with heart failure.

## 6 Conclusion

In patients with DCM, QRS-prolongation, with or without LBBB pattern, is frequently present and often co-exists with septal midwall LGE on CMR. Both markers are significantly associated with structural LV changes in myocardial remodelling. However, they are not directly correlated. This suggests assessment of both QRS duration and septal midwall LGE on CMR to be of complementary value for risk-stratification o patients with DCM.

## CRediT authorship contribution statement

**Marthe A.J. Becker:** Conceptualization, Data curation, Investigation, Formal analysis, Methodology, Project administration, Visualization, Writing - original draft. **Cornelis P. Allaart:** Conceptualization, Resources, Software, Supervision, Writing - review & editing. **Alwin Zweerink:** Conceptualization, Investigation, Writing - review & editing. **Jan H. Cornel:** Resources, Supervision, Writing - review & editing. **Peter M. Ven:** Methodology, Formal analysis, Writing - review & editing. **Albert C. Rossum:** Resources, Supervision, Writing - review & editing. **Tjeerd Germans:** Conceptualization, Methodology, Supervision, Visualization, Writing - review & editing.

## Declaration of Competing Interest

No conflict of interest. No grants or other form of financial support received.
